# Assessment of mismatch repair deficiency in ovarian cancer

**DOI:** 10.1136/jmedgenet-2020-107270

**Published:** 2020-09-11

**Authors:** Emma J Crosbie, Neil A J Ryan, Rhona J McVey, Fiona Lalloo, Naomi Bowers, Kate Green, Emma R Woodward, Tara Clancy, James Bolton, Andrew J Wallace, Raymond F McMahon, D Gareth Evans

**Affiliations:** 1 Division of Cancer Sciences, The University of Manchester, Manchester M13 9WL, UK; 2 Department of Obstetrics and Gynaecology, St Mary's Hospital, Manchester University NHS Foundation Trust, Manchester Academic Health Science Centre, Manchester M13 9WL, UK; 3 Division of Evolution and Genomic Medicine, The University of Manchester, Manchester M13 9WL, UK; 4 Department of Pathology, Manchester University NHS Foundation Trust, Manchester Academic Health Science Centre, Manchester M13 9WL, UK; 5 Manchester Centre for Genomic Medicine, North-West Genomics Laboratory Hub, Manchester University NHS Foundation Trust, Manchester Academic Health Science Centre, Manchester M13 9WL, UK

**Keywords:** genetic testing, surgical oncology, genetic predisposition to disease, gynecology

## Abstract

**Background:**

Hereditary causes of ovarian cancer include Lynch syndrome, which is due to inherited pathogenic variants affecting one of the four mismatch repair genes involved in DNA repair. The aim of this study was to evaluate tumour mismatch repair deficiency and prevalence of Lynch syndrome in high-risk women referred to the Manchester Centre for Genomic Medicine with ovarian cancer over the past 20 years.

**Methods:**

Women with ovarian cancer diagnosed before the age of 35 years and/or with a suggestive personal or family history of Lynch syndrome cancers underwent tumour testing with immunohistochemistry for mismatch repair deficiency and, where indicated, *MLH1* promoter methylation testing followed by constitutional testing for Lynch syndrome.

**Results:**

In total, 261 ovarian cancers were tested and 27 (10.3%; 95% CI 6.9% to 14.7%) showed mismatch repair deficiency by immunohistochemistry. Three of 7 with MLH1 loss showed *MLH1* promoter hypermethylation, and 18 of the remaining 24 underwent constitutional testing for Lynch syndrome. A further 15 women with mismatch repair proficient tumours underwent constitutional testing because of a strong family history of Lynch syndrome cancers. Pathogenic variants were identified in 9/33 (27%) women who underwent constitutional testing, aged 33–59 years (median 48 years), including one whose tumour was mismatch repair proficient. Most Lynch syndrome tumours were of endometrioid histological subtype.

**Conclusions:**

Tumour mismatch repair deficiency identified by immunohistochemistry is a useful prescreen for constitutional testing in women with ovarian cancer with personal or family histories suggestive of Lynch syndrome.

## Introduction

Ovarian cancer is the seventh most common malignancy worldwide and the most lethal gynaecological cancer.^1–3^ Epithelial ovarian cancer is one of the most heritable malignancies, frequently due to pathogenic variants in single high-risk genes. The heritable component of ovarian cancer is predominantly due to constitutional pathogenic variants in *BRCA1* and *BRCA2* with as many as 22% of women with high grade serous ovarian cancers (HGSOC) carrying pathogenic variants in these genes.^4^ The other leading heritable cause is Lynch syndrome (LS), an inherited mismatch repair (MMR) deficiency due to constitutional pathogenic variants affecting one of the four MMR genes, *MSH2, MLH1, MSH6* and *PMS2*.^5^ Around 1:280 of the general population carries a pathogenic variant in a MMR gene, the great majority of whom are undiagnosed.^6^ Women heterozygous for pathogenic MMR gene variants have a 3%–17% lifetime risk of ovarian cancer, and higher risks for colorectal and endometrial cancers.^7 8^

Since the discovery of the MMR genes in 1993–1994, clinicians have tried to target constitutional testing for LS to those at highest risk. The Amsterdam criteria were developed in 1991,^9^ but these require a strong family history of colorectal cancer to be discriminatory. Even adding additional LS tumours to the criteria, such as endometrial and ovarian cancer^10^ has added little to its detection rate^11^ or sensitivity.^12 13^ Testing for LS outside of the Amsterdam criteria,^9 10^ where upfront constitutional testing is practised, has largely depended on a prescreen of the incident tumour using immunohistochemistry (IHC) for MMR protein expression or DNA for microsatellite instability (MSI).^12^ There have been very few studies that have looked at the success of this prescreen in ovarian cancer and most have included small numbers of tumours and have concentrated on just one histological subtype of ovarian cancer (endometrioid).^13 14^ This ignores the fact that restricting testing based on histological subtype misses cases of LS, particularly as morphology is subjective and can be challenging in complex cases.^15 16^

We have evaluated our prescreening strategy with IHC in women referred to the regional genetics department with possible LS-associated ovarian cancer from 2000 to 2020 and assessed the identification of constitutional MMR pathogenic variants.

## Methods

### Participants

Women referred to the regional genetics department in Manchester with ovarian cancer and concerns about the possibility of LS provided consent for tumour and if indicated constitutional testing. Most women had a history of another LS-related cancer in themselves or another family member (colorectal, endometrial, ovary, biliary tree, urinary tract, gastric or skin). However, some were selected based on diagnosis at <35 years of age.

### Immunohistochemistry

IHC for the four MMR proteins was performed in the clinical pathology laboratory using the automated Ventana BenchMark ULTRA IHC⁄ISH staining module and the OptiView, 3’diaminobenzidine V.5 detection system (Ventana, USA) according to standard clinical protocols. Tumour epithelial MMR expression was scored by two expert independent observers using stroma as internal control and as described previously.^17^

### Methylation analysis

Reflex *MLH1* promoter methylation testing was performed on tumours showing loss of MLH1 on IHC. Extracted DNA was bisulfite converted and then amplified with bisulfite specific primers in triplicate. A region of the *MLH1* promoter containing four CpG dinucleotides whose methylation status is strongly correlated with MLH1 expression were sequenced using a pyrosequencer (PSQ 96MA). Two independent scientists interpreted the pyrograms. ‘Hypermethylation’ was described as >10% mean methylation across the four CpG dinucleotides on a minimum of two of three replicate analyses. In addition to promoter methylation analysis, testing was carried out for the *BRAF* c.1799T>A variant in some cases.

### Microsatellite instability analysis

Extracted DNA underwent sodium bisulfite conversion using the Epitect Plus FFPE kit (Qiagen, UK). The MSI analysis system V.1.2 (Promega, USA) used fluorescent-labelled primers to coamplify seven markers, including five mononucleotide-repeat markers (BAT-25, BAT-26, NR-21, NR-24 and MONO-27), and two control penta-nucleotide-repeat markers (Penta-C/Penta-D). MSI status was reported as microsatellite stable (MSS) where all five mononucleotide loci between tumour and matched normal tissue were identical; MSI-low (MSI-L) where there was discordance in one mononucleotide locus and MSI-high (MSI-H) where two or more mononucleotide loci were discordant.

### Constitutional analysis

DNA was extracted from 2 to 5 mL lymphocyte blood (EDTA anticoagulant) using Chemagic DNA blood chemistry (CMG-1097-D) on an automated Perkin Elmer Chemagic 360 Magnetic Separation Module and a JANUS Integrator 4-tip Automated Liquid handling platform. DNA was eluted into 400 uL buffer. Extracted DNA samples were measured for DNA yield, concentration and quality using a Nanodrop ND-8000 spectrophotometer. Three MMR genes *MLH1*, *MSH2* and *MSH6* were amplified using long range PCR followed by next generation sequencing using Illumina SBS v2 2×150 bp chemistry on an Illumina MiSeq. The whole coding region, intronic flanking sequences to±15 bp and known splicing variants of *MLH1*, *MSH2* and *MSH6* were analysed (minimum 100 x coverage depth). Variant identification and calling was via an in-house bioinformatic pipeline. Reported sequence changes and regions with <100× coverage were retested via Sanger sequencing using BigDye V.3.1 chemistry. Copy number analysis to detect large genomic rearrangements affecting the three MMR genes was performed using MLPA MRC-Holland probe mixes: P003-D1 *MLH1*/*MSH2* and P072-C1 *MSH6*. Variant nomenclature followed Human Genome Variation Society guidelines (http://www.hgvs.org/vamomen) using reference sequences: LRG_216, t1(MLH1); LRG_218, t1(MSH2); LRG_219, t1(MSH6). Exons were numbered consecutively starting from exon 1 as the first translated exon for each probe mix. Cases with PMS2 protein loss, normal *MLH1* methylation and no path_*MLH1*/*MSH2*/*MSH6* variant underwent path_*PMS2* analysis at the regional specialist Yorkshire and North East Genomic Laboratory.

All women gave written informed consent for tumour and blood testing except deceased cases, whose tumour was obtained and tested with a relative’s consent. Advice from our ethics committee was that the current analysis represented clinical service evaluation and that no specific ethics application was required. There is no directly identifiable patient information presented.

### Statistics

Differences between values were tested by a two-tailed Fisher's χ^2^ test.

## Results

**Figure 1:**
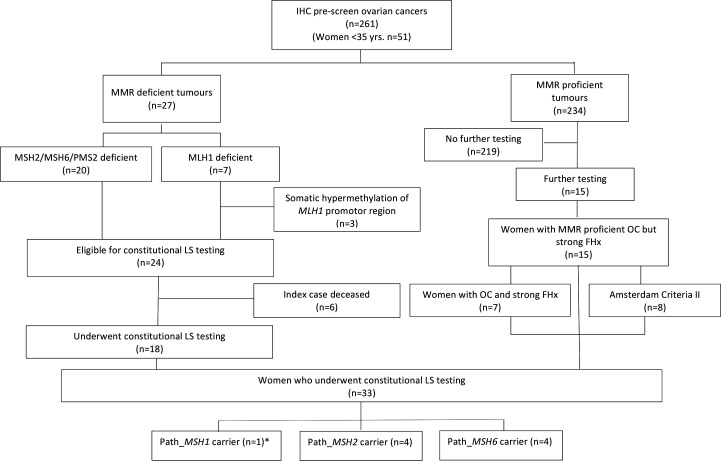
Study flow diagram. *Includes LS carrier found in a MMR proficient case with a simultaneous constitutional pathogenic variant in *BRCA 1*. FHx, family history; IHC, immunohistochemistry; MMR, mismatch repair; LS, Lynch syndrome; OC, ovarian cancer.

In total, 261 women with ovarian cancer underwent an IHC prescreen for LS (table 1, figure 1). They were aged between 16 and 89 years (median=49 years). Fifty-one cases were tested because they were diagnosed at <35 years of age. All histological subtypes were tested if indicated, with HGSOC the most frequently tested. Overall, only 27 (10.3%; 95% CI 6.9% to 14.7%) tumours showed MMR deficiency by IHC with just 7 (2.7%) having loss of MLH1 (table 2). Three of these tumours showed *MLH1* promotor hypermethylation and therefore constitutional LS testing was not performed. Eighteen of the remaining 24 women whose tumours showed MMR deficiency underwent constitutional testing for MMR pathogenic variants. The remaining six did not undergo constitutional analysis because the ovarian cancer case was deceased and a blood lymphocyte sample was not available. An additional 15 women underwent constitutional analysis despite having MMR proficient tumours due to a strong family history, with eight meeting Amsterdam II criteria^9^ (figure 1).

**Table 1:** Number of ovarian cancers tested for IHC by pathology and proportion of women with MMR pathogenic variants

Pathology	Tested (n)	IHC loss (n)	IHC loss (%)	BRAF tested (n)	C.1799T>A positive (n)	C.1799T>A positive (%)	Methylation tested (n)	Hypermethylated (n)	Hypermethylated (%)	Tested for path_MMR (n)	Lynch syndrome (n)	Lynch syndrome (%)	
Endometrioid	43	9	20.9	2	0	0	4	2	50	10	4	40	*MSH2,* *3 MSH6*
Clear cell	19	2	10.5	0	–	–	0	–	–	4	2	50	*MLH1, MSH2*
Mucinous	59	6	10.2	1	0	0	1	0	0	10	0	0	–
Low grade serous	10	0	0	0	–	–	0	–	–	0	0	0	–
High grade serous	79	6	7.6	1	0	0	1	0	0	9	2	22	*MSH2, MSH6*
Adenocarcinoma (other)	38	3	7.9	1	0	0	1	1	100	2	0	0	–
Other*	13	1	7.7	0	–	–	0	–	–	1†	1	100	*MSH2*
Total	261	27	10.3	5	0	0	7	3	43	36	9	25	*4 MSH6, 4 MSH2, 1 MLH1*

*Three Mullerian, two granulosa cell, one Sertoli, two secondaries, one mesodermal, one Brenner, three carcinosarcoma.

†Carcinosarcoma of ovary aged 48 years; sister had colorectal cancer aged 34 years.

IHC, immunohistochemistry; MMR, mismatch repair.

**Table 2:** IHC loss and constitutional MMR pathogenic variant detection rates in all index ovarian cases tested

	Tested (n)	IHC loss (n)	IHC loss (%)	Tested for path_MMR (n)	Lynch syndrome (n)	Lynch syndrome (%)
Any loss	261	27	10.3	18	8	44.4
MLH1 loss	261	7	2.7	3	0	0.0
Either MSH2 or MSH6	261	19	7.3	15	8	53.3
MSH2 loss	261	13	4.9	9	4	44.4
MSH6 loss	261	10	3.8	9	6	66.7
MSH6 loss alone	261	7	2.7	6	4	66.7
PMS2 loss alone	261	0	0.0	0	0	–
No Loss	234	0	0.0	15	1*	6.7

*Ovarian clear cell carcinoma aged 59 years had exon 6–19 deletion of *MLH1* with normal IHC –family met Amsterdam II criteria. She also carries a *BRCA1 exon 13* duplication. She developed grade 3 triple negative breast cancer at 71 and sebaceous carcinoma at 67 years.

IHC, immunohistochemistry; MMR, mismatch repair.

MSI testing was performed for 24/261 cases. Five tumours were MSI-H, all of which were MMR deficient by IHC and 4/5 women underwent constitutional analysis for MMR pathogenic variants. Two tumours were MSI-L and MMR deficient, and one out of two of these women underwent constitutional analysis. Seventeen tumours were MSS, six of which were MMR deficient and five of these six women underwent constitutional analysis for MMR pathogenic variants. Four of six path_MMR carriers had MSI-H tumours; one was MSI-L (*MSH2*), one MSS (*MSH6*) and the remaining three were not MSI tested (table 3).

**Table 3:** Ovarian cancer cases with Lynch syndrome

Gene	FIGO stage and histological subtype	Age (years)	IHC loss (4 protein panel)	MSI	Type of pathogenic variant	Path_MMR variant	Meets Amsterdam criteria?
*MLH1*	Stage 1c clear cell	59	None	Not tested	Large rearrangement	*MLH1* exon 6–19 deletion	Yes—Amsterdam modified
*MSH2*	Stage 1a mixed endometrioid/clear cell	34	MSH2 loss	MSI-H	Splice site	*MSH2* c.1276+2T>C	No
*MSH2*	Stage 3c high grade serous	38	MSH2 and MSH6 loss	MSI-L	Truncating	*MSH2* c.528_529delTG	No
*MSH2*	Stage 1a carcinosarcoma	48	MSH2 and MSH6 Loss	MSI-H	Large rearrangement	*MSH2* exon 3 deletion	No
*MSH2*	Stage 1c endometrioid	51	MSH2 and MSH6 loss	MSI-H	Truncating	*MSH2* c.196delT	No
*MSH6*	Stage 1a endometrioid	47	MSH6 loss	Not tested	Splice site	*MSH6* c.3439–1G>T	Yes—Amsterdam modified
*MSH6*	Stage 1 endometrioid	50	MSH6 loss	MSI-H	Missense	*MSH6* c.1346T>C p.Leu449Pro	No
*MSH6*	Stage 2 high grade serous	50	MSH6 loss	MSI-S	Truncating	*MSH6* c.3732_3735dupATTT	No
*MSH6*	Stage 3c poorly differentiated endometrioid with focal neuroendocrine features	53	MSH6 loss	Not tested	Truncating	*MSH6* c.3261delC	No

IHC, immunohistochemistry; MMR, mismatch repair.

MMR pathogenic variants were found in 9/33 (27%) women who underwent constitutional testing with ages of ovarian cancer diagnosis of 33–59 years (median 48) (table 3). The highest rate was for endometrioid ovarian cancer with 10/43 (9.6%) having a constitutional MMR pathogenic variant. The only LS case whose ovarian tumour was MMR proficient was a patient with a clear cell carcinoma who also had a constitutional *BRCA1* pathogenic variant. It is therefore likely her ovarian cancer was not driven by her *MLH1* pathogenic variant. There were four pathogenic variants each in *MSH2* and *MSH6,* the *MSH6* cases only displayed MSH6 IHC loss whereas three of the *MSH2* pathogenic variants had loss of both MSH2 and MSH6. Selection for ovarian cancer by age <35 years was not effective as a selection tool as only 4/52 (7.7%) had IHC loss. Only one of the four women<35 years tested for constitutional pathogenic variants had a path_MMR variant identified, and that patient had a parent with four separate bowel primary tumours highly suggestive of LS (table 3).

## Discussion

Here, we describe our 20-year experience of tumour MMR IHC as a prescreen for constitutional testing women with suspected LS-associated ovarian cancer. We tested 261 ovarian tumours for MMR deficiency because women were diagnosed <35 years of age and/or because they had a suggestive personal or family history of LS. Those with strong clinical risk factors underwent constitutional testing even if their tumours were MMR proficient. In total, 27 tumours (10.3%) were MMR deficient and 8 of these had LS. Most were of endometrioid histological subtype. One woman with constitutional path_MMR variant had a MMR proficient tumour; she also had a constitutional *BRCA1* pathogenic variant. She is unlikely therefore to have developed ovarian cancer via a MMR driven pathway.

Previous studies examining the MMR status of unselected endometrioid or clear cell ovarian cancers found similar rates of MMR deficiency, but overall numbers were very small.^13 14 18^ Two systematic reviews found that approximately 10% of ovarian tumours are MMR deficient by IHC, but included studies that were very limited with respect to their reporting of basic epidemiological, molecular and clinical features.^19 20^ There was also poor reporting of constitutional status. A recent study by Leskela *et al* 21 examined the MMR status of 502 stage I/II tumours selected from the GEICO Early Stage Ovarian Cancer Registry. The authors report MMR deficiency in 18.7% endometrioid and 2.4% clear cell tumours overall, but do not provide information about clinical risk factors for LS in their cohort. It is perhaps surprising that despite selecting for LS features, such as early age of cancer onset and indicative personal or family history, that detection rates were not higher in our study, with only 10.3% with IHC loss and 3.5% with a path_MMR variant. Age selection (<35 years) was not an effective triage strategy for constitutional testing with only 7.7% IHC loss and 1.9% path_MMR. Furthermore, failure to select for pathology type by including serous histological subtypes will have further reduced our detection rates.

There are several strengths to our work. First, we carried out MMR IHC tumour prescreening for all women referred to the clinical genetics department whose age and family history were suggestive of LS-associated ovarian cancer. We did not restrict testing to any particular histological subtype. This is important because histological subtyping is subjective, challenging in difficult cases and has evolved considerably over the past 20 years, with validated IHC panels increasingly used to assist diagnosis. Many of our cases pre-dated the now gold standard expert gynaecological pathology review and confirmation by IHC.^16^ Restricting testing to endometrioid subtype would deny LS testing to women with ovarian cancer diagnosed and treated historically and in non-expert centres. In particular, women diagnosed with non-endometrioid tumours who have survived without recurrence from this earlier era may harbour a constitutional MMR pathogenic variant, as survival in LS is known to be good, and tumours may on review be reclassified with modern pathology.^15^ Second, we provide detailed clinical annotation for all proven LS-associated ovarian tumours as well as comprehensive molecular phenotyping, including MMR, MSI and, where indicated, *MLH-1* promoter methylation status. Analyses were carried out to quality-assured clinical standards in specialist pathology and genetics referral laboratories. Data were collected from our prospective clinical database, ensuring comprehensive reporting of all cases and minimising issues with missing data. All non-deceased women with MMR deficient ovarian tumours unexplained by *MLH1* promoter hypermethylation and 15 others, whose clinical risk factors were particularly suggestive, underwent definitive constitutional LS testing using blood lymphocyte DNA. This compares favourably with preceding series where the conversion to constitutional testing was poor and pathogenic variants were assumed from allele frequency in adjacent normal tissue.^18–21^ Third, we tested 15 women with strong clinical risk factors whose ovarian tumours were MMR proficient, facilitating an assessment of the accuracy of MMR IHC as a prescreen for LS constitutional testing, which is poorly reported in the literature. We found only one case of MMR proficient LS-associated ovarian cancer, in a woman who also carried a *BRCA1* pathogenic variant and whose tumour is likely to have developed via a non MMR driven pathway.

Limitations of the study include failure to conduct MSI analysis for all cases, which precludes a direct comparison between MMR IHC and MSI status as a prescreen for constitutional LS testing. The single centre nature of this study is another limitation, since we cannot necessarily extrapolate our conclusions to other healthcare settings where clinical genetics referral criteria for suspected LS may differ. Our cohort was selected for IHC testing and downstream analyses based on clinical criteria and therefore may not reflect the MMR status of unselected ovarian cancer populations.

The emergence of targeted therapies has led to mainstream somatic and/or constitutional *BRCA1/2* sequencing of women with ovarian cancer to inform suitability for PARP inhibitor therapy and clinical trial enrolment.^22 23^ Given the similar cumulative risk of ovarian cancer in LS to *BRCA2*, testing premenopausal women with epithelial ovarian cancer for both *BRCA1/2* and LS is appropriate, particularly in an era of panel gene testing where there is little additional cost to add more genes.^24^ If this practice becomes widespread, it may reduce the requirement for a prescreen for LS testing of patient with recently diagnosed ovarian cancer, although a prescreen would still be useful for women referred to clinical genetics departments with a previous history of ovarian cancer, in whom *a priori* panel gene somatic testing is unlikely to be indicated.

In summary, we report our experience of MMR IHC as a prescreen for constitutional MMR pathogenic variant testing in women with clinical risk factors for LS-associated ovarian cancer. LS is rare if tumours are MMR proficient. While most LS-associated ovarian tumours are of endometrioid histological subtype, the subjective and sometimes challenging task of pathological interpretation risks misclassification. Thus, our practice is to continue to prescreen all ovarian tumours with clinical risk factors for LS irrespective of tumour histological subtype, especially if their tumour pre-dates recent multidisciplinary panel review in an expert centre.

## Data Availability

Data are available from the corresponding author on reasonable request.
